# Sex-Specific Differences in Nutritional Status and Olfaction in Association with Cognitive Impairment Amongst Older Adults with Long COVID Syndrome

**DOI:** 10.3390/jcm15051994

**Published:** 2026-03-05

**Authors:** Alma L. Guzmán-Gurrola, Laura González-López, Jonathan S. Chávez-Íñiguez, Mariana Verduzco Vázquez, Efraín I. Flores-Hernández, José A. Novoa-Burquez, Maria G. Zavala-Cerna

**Affiliations:** 1 High Specialty Geriatric Care Unit, Hospital Civil de Guadalajara Fray Antonio Alcalde, Guadalajara 44200, Mexico; aguzman@hcg.gob.mx; 2 Centro Universitario de Tlajomulco, Universidad de Guadalajara, Tlajomulco 45641, Mexico; 3 Research Department, Universidad del Valle de Atemajac Campus Guadalajara, Guadalajara 45050, Mexico; laura.gonzalez@univa.mx; 4 Nephrology Department, Hospital Civil de Guadalajara Fray Antonio Alcalde, Guadalajara 44200, Mexico; jschavez@hcg.gob.mx; 5 Centro Universitario Ciencias de la Salud, Universidad de Guadalajara, Guadalajara 44340, Mexico; 6 Immunology Research Laboratory, Decanato de Medicina, Universidad Autónoma de Guadalajara, Guadalajara 45129, Mexico; mariana.verduzco@edu.uag.mx; 7 Decanto Ciencia y Tecnología, Universidad Autónoma de Guadalajara, Guadalajara 45129, Mexico; efrain.flores@edu.uag.mx; 8 Instituto de Seguridad y Servicios Sociales de los Trabajadores del Gobierno y Municipios del Estado de Baja California, Tijuana 22520, Mexico; anovoa@uabc.edu.mx

**Keywords:** olfactory capacity, nutritional risk, mini nutritional assessment, cognitive impairment, post-COVID syndrome, older adults

## Abstract

**Background/Objectives**: Long COVID has emerged as a significant public health concern, characterized by persistent symptoms following SARS-CoV-2 infection. Cognitive impairment is a common sequela, particularly among older adults (OAs). Although olfactory dysfunction and malnutrition have been previously associated with cognitive decline, it remains elusive to what extent sex-specific variations in these and additional factors will be pivotal to guiding targeted interventions in a sex-specific manner. To fill this gap in knowledge, we undertook a study with the purpose of investigating the contribution of sex-specific risk factors to the development of cognitive impairment (CI) in a cohort of OAs hospitalized with long COVID. **Methods**: We undertook a cross-sectional study among OAs hospitalized at a geriatric care unit. Olfactory function was assessed using the Sniffin’ Stick Test. Cognitive impairment was evaluated by the Mini-Mental State Examination, and nutritional status was assessed with the Mini Nutritional Assessment (MNA). Statistical analyses included linear regression. **Results**: A total of 45 patients with long COVID were included, of whom 51% were female. The prevalence of CI was lower in men compared to women. In the single variable analysis, nutritional factors were associated with CI only in women; importantly, the loss of olfactory function was associated with CI in the whole group and to CI in women after multivariate analysis. **Conclusions**: Olfactory dysfunction is a potential biomarker for cognitive impairment in OAs with long COVID in a sex-specific manner. In our study nutritional status and probable obesity could be additional factors associated with CI; nevertheless, this was not confirmed in our multivariate analysis; therefore, this hypothesis would need to be tested in larger studies.

## 1. Introduction

Long COVID has emerged as a health problem worldwide; it is a systemic condition [1] defined by a persistence in symptoms after an acute confirmed infection with SARS-CoV-2 for at least three months as a continuous, relapsing/remitting, or progressive disease state [2] with an incidence and prevalence of 50% and 45% respectively [3]. Common signs and symptoms include fatigue, the loss of memory, cognitive impairment (CI), difficulty breathing, thoracic pain, anosmia, ageusia, arthralgias, myalgias and functional impairment [4].

One of the most distinctive symptoms is the loss of olfactory capacity, affecting 85 to 98% during the acute infection and up to 80% during long COVID [5,6,7,8]. The prevalence of olfactory changes in older adults (OAs) is 27% and occurs more frequently in men [9].

Multiple investigations into long COVID have shown sex-specific risk factors, such as a higher prevalence and higher rates of persistent symptoms in women [10,11]. Specific symptoms found to be more frequent in women include fatigue, dyspnea, physical pain, hair loss, ocular disturbances, depression, and/or sleep disorders [12]. This gender bias is also reflected in neurological and psychiatric manifestations, with women more frequently reporting headaches, sleep disturbances, and CI compared to men [13].

The development of CI is of special interest in OAs, since they are a vulnerable population due to defects in immune response, typically associated with a high prevalence of cardiorenal metabolic, and geriatric comorbidities; as a result, OAs have an increased risk of CI [14] and malnutrition [15]. CI has been a common neuropsychiatric consequence of COVID infection [16,17,18], believed to appear as a result of increased permeability in the encephalic barrier and irreversible neuronal damage [19], with a deterioration in memory function, microvascular endothelial dysfunction, the presence of inflammatory metabolites and beta-amyloid accumulation within the brain [20]. Women often have a higher prevalence of cognitive impairment and dementia compared to men [21]. Other studies have shown that women can experience a faster cognitive decline compared to men in global cognition and executive function [22]; however, systematic reviews and larger cohort studies suggest that major differences related to sex in CI emerge after age 80 in specific domains [23]. Some biologic and social factors have been suggested to account for these differences, such as lower education and social disadvantage [24]; other differences, typically associated with aging, have not been properly addressed in a sex-specific manner, such as malnutrition, obesity, and the loss of olfactory function [25,26]. Based on prior evidence suggesting sex-related differences in nutritional vulnerability, olfactory dysfunction, and cognitive outcomes after SARS-CoV-2 infection, we hypothesize that nutritional status, olfactory function, and inflammatory biomarkers can be associated in a sex-specific form to cognitive impairment in OAs with long COVID syndrome to guide better-targeted treatments. To fill this gap in knowledge, we undertook a cross-sectional study with the purpose of investigating the contribution of sex-specific risk factors to the development of CI in hospitalized OAs with long COVID.

## 2. Results

During the study period, a total of 90 patients were hospitalized in our clinic with a diagnosis of long COVID. The main reasons for hospitalization were: upper respiratory tract infection (38%), cerebrovascular or neurological infections (29%), general malaise/fever of unknown origin (15%), GI tract infection/dehydration (13%), and anemia or cardiometabolic alterations (4%).

**Figure 1:**
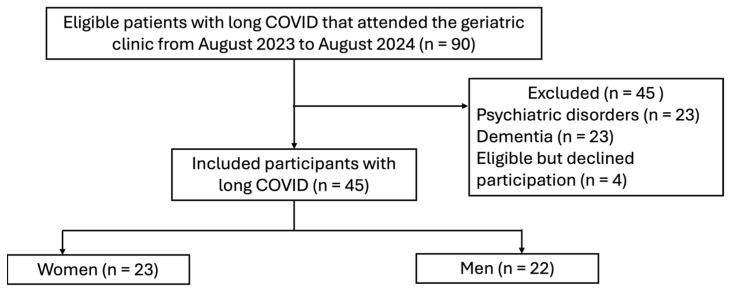
Study flow diagram. CI: cognitive impairment.

For all hospitalized patients, eligibility was assessed, and subjects that met the inclusion criteria were invited to participate in the study (Figure 1).

Overall, the studied population had an age range from 65 to 89 years old, and 51% of the studied population were female. The more frequent comorbidities included hypertension in 55.6%, Type 2 diabetes (T2D) in 40%, Chronic Obstructive Pulmonary Disease (COPD) in 35.6% and chronic kidney disease (CKD) in 22.2%; more than 50% of the studied population had more than three comorbidities at the time of the assessment. With respect to the nutritional status, a total of 23 participants (51%) were in the normal/risk group, and 22 (49%) had malnutrition.

During the acute COVID infection, up to 53.3% of participants required hospitalization, with a median (ICR) of 5 (0–15) hospital days. Therapy with supplementary oxygen was required by 75.5% and 4% required assisted mechanical ventilation. In the post-COVID stage, 77.8% were unable to wean off supplemental oxygen, 86% presented anosmia, 91.1% fatigue, 55.5% ageusia, 73.3% memory problems, and 77.7% insomnia. The average duration of these symptoms was 99.48 ± 128.15 days.

With respect to the sex-specific analysis in the studied population, the prevalence of CI was lower in men 13 (59.1%) than in women 17 (73.9%), while the prevalence of malnourishment was equal at 18 (50), although MNA was slightly lower in women at 17.0 (15.5–23) compared to men at 18.5 (15.5–22.5) (*p* = 0.865) (Table 1).

To analyze factors associated with CI, we performed a sub-analysis in a sex-specific manner for CI; we observed that women with a wider calf circumference and mid-arm circumference, probably associated with the presence of obesity, as well as a higher MNA score, were significantly associated with CI; whereas in men, anthropometric measurements were not found in association with CI (Table 2).

With respect to long COVID, we found that symptoms such as ageusia, memory problems, depression and myalgias were more frequent in women compared to men, although only the presence of memory problems was significantly associated with CI for both sexes; additionally, palpitations and brainfog were significantly associated with CI only in men, while a larger number of days with long COVID was significantly associated with CI exclusively in men (Table 2).

During the acute infection, men appear to have experienced more severe COVID-19, with a higher number of hospitalizations and a higher need for supplementary O_2_ compared to women. With respect to biochemical parameters, we found that a lower platelet count was significantly associated with CI exclusively in women. The remaining parameters were non-significantly associated with CI. When analyzing comparisons only between men and women affected by CI, we found that men with CI had a significantly lower BMI (23.3 ± 4.5) compared to women with CI (29.5 ± 6.3) (*p* < 0.0051), a smaller calf circumference (29.4 ± 4.1) in comparison to women (33.0 ± 3.9) (*p* = 0.0204) and a smaller mid–upper arm circumference (25.5 ± 3.8) compared to women with CI (30.2 ± 5.5) (*p* = 0.0177).

Then, to address for confounding, we performed a multivariate analysis in a sex-specific manner (Table 3). Variables included in the final model were selected from the results of single variable regressions, those with biological plausible contribution, and variables from our original hypothesis. In our final model, the only variable that remained significantly associated with CI was the sniffing test for women; for every increased point in the MMSE, the sniffing test increases 1.664 (*p* = 0.009).

**Figure 2:**
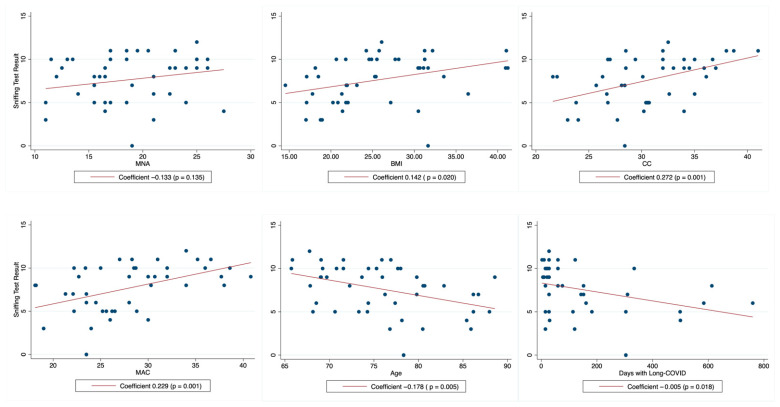
Fitted graphs for regressions between sniffing test and continues variables related to nutritional status, age and days with long COVID. MNA: Mini Nutritional Assessment, BMI: body mass index, CC: calf circumference, and MAC: mid-arm circumference.

With respect to the olfactory capacity, 53.3% of the studied population had hyposmia and 33.3% had anosmia. We found that the olfactory capacity, assessed by the sniffing test, had a positive correlation with MMSE (*p* = 0.012) (Figure 2), and a negative correlation with increased age (*p* = 0.005) and the number of days with long COVID (*p* = 0.018). Interestingly, the sniffing test was not significantly associated with MNA, but it showed a significant association with CC, MAC, BMI, and obesity (Table 4).

Then, we tested variables associated with an altered sense of smell, adjusting for confounding in a sex-specific manner with multiple variable regressions (Table 5), where only the number of days with long COVID was significantly associated with an altered sense of smell for women and not men.

## 3. Discussion

In this cohort of OAs with long COVID, cognitive impairment (CI) was identified in more than two-thirds of study participants. In previous studies, persistent inflammation during long COVID had been associated with CI and behavioral disorders [27]. We found several measurements of the nutritional state such as BMI, CC and MAC to be associated with either cognitive decline or an altered sense of smell, but this was not confirmed in multiple variable analysis. After adjusting for confounding, the only variable that remained significantly associated with CI was the sniffing test for women, and the number of days with long COVID with an altered sense of smell, also in women. For men, no variable remained significantly associated with CI or an altered sense of smell.

Half of the study participants presented with malnourishment and were analyzed with the MNA test, which has been very useful in identifying the risk of malnourishment but does not consider other abnormalities such as obesity or overweight in comparison to other measurements, such as the NRS-2002, MUST and SGA [28]. Diagnosing malnutrition may be complicated, since obesity may mask malnourishment [29], which is relevant to our study, considering that obesity and overweight have been previously associated with COVID infection [30]. In our analyzed participants, obesity was more frequent in women, and was associated with CI exclusively in women, but only during single variable analysis; the hypothesis that obesity could be an independent risk factor for CI in long COVID would need to be tested in larger cohorts, after adjusting for confounding. The MNA test showed no association with CI or an altered sense of smell in multiple variable regression analysis for either women or men.

Recent studies have consistently highlighted sex-related differences in the prevalence and clinical expression of long COVID. In our study, men presented more frequently self-reported palpitations and fatigue, with the remaining symptoms being presented in similar frequencies for both sexes. The number of days with long COVID was slightly higher for men compared to women. Previously observed differences in symptoms and clinical characteristics during long COVID have been previously associated with hormonal and immunological factors, particularly estrogen-mediated immune modulation, which may contribute to the heightened vulnerability of women to long COVID syndrome [31]. We did not include in our study hormonal or immunological factors; further mechanistic studies are required to confirm this relationship. In our study, men appear to experience more severe acute COVID-19, with a higher number of hospitalizations and a higher need for supplementary O_2_, which is consistent with previous studies reporting that men have more severe cases with reportedly higher mortality rates [11]. Importantly, the consistency of these systematic results across diverse populations suggests that sex differences in long COVID risk are not fundamentally due to cultural or healthcare access disparities, but rather reflect an underlying biological and pathophysiological mechanism contributing to the outcome [12].

The sense of smell is essential as it stimulates appetite, promotes food intake, and is associated with a better quality of life [32]. Anosmia is considered a common and cardinal symptom of COVID-19, particularly in the early stages of infection, sometimes being the only symptom in an otherwise asymptomatic subject. SARS-CoV-2 primarily targets cells expressing the angiotensin-converting enzyme 2 (ACE2) receptor, which is abundantly present in the nasal epithelium, particularly on non-neuronal cells. Key cells involved in the loss of olfactory function are the sustentacular cells that support the function of olfactory neurons [33,34]. SARS-CoV-2 infection reduces the olfactory capacity through multiple pathways, including endothelial dysfunction, autoimmunity, latent viral reactivation, hyperinflammation, and autonomic nervous system dysfunction [35]. Importantly, as the olfactory capacity decreases, the risk of malnutrition increases [36]. In our study, more than two-thirds of patients had hyposmia or anosmia, and as the olfactory capacity worsens, punctuations in the MMSE decline—a similar finding to previous studies [37]. The association of impaired olfaction and cognitive decline might be partially explained by the previously described pathophysiological mechanism for olfactory dysfunction during COVID infection, which includes immune hyperactivation, neuroinflammation, direct viral encephalitis, blood–brain barrier integrity damage, hypoxia, and cerebrovascular disease [32], all of which can be associated with CI as well. Additionally, to add layers of complexity, aging plays a fundamental role in the olfactory capacity, reaching its peak at age 40 and afterwards progressively declining [26], another factor associated with cognitive decline. In our study, increased age was not significantly associated with CI; however, increased age was significantly associated with impaired olfaction measured by the sniffing test.

When we analyzed olfaction in women and men, we noticed that in a multiple variable analysis the only factor significantly associated with an altered sense of smell was the number of days with long COVID, and it was only found in women. Other variables were non-significantly associated in a sex-specific manner, probably due to the small group for each sex. Furthermore, it has been estimated that olfactory impairment could be more common in men compared to women [38]. In our study, olfactory impairment was slightly more frequent in men, but it was not significantly associated with CI nor MNA score.

A similar study to ours evaluated the relationship between olfactory dysfunction, nutritional status, and cognitive function in 45 geriatric patients with neurodegenerative diseases and found that olfactory function had no association with nutritional status [39]. This cohort differs from ours, as it was conducted in 2016, before the emergence of COVID-19; therefore, distinct pathophysiological mechanisms for olfactory decline were involved.

Some of the limitations of our study include the cross-sectional design, which is not suitable to imply causality but rather to generate hypotheses. The sample size is small, especially for stratified analysis by sex, and there are multiple confounders that can participate in the generation of CI. We did not measure endocrine or inflammatory biomarkers, nor did we include information about treatments during the acute infection or for long COVID. Likewise, the information about the viral strain infecting individuals was not available for the study population; therefore, incomplete information related to the acute infection could lead to confounding that was not addressed by this study. Testing or formal functional evaluation of cognitive decline was not reassessed in participants involved in the study; therefore, mis-classification bias cannot be excluded.

The strengths of our study lie in its population: older adults with long COVID, who have been an understudied population despite suffering from severe symptoms. We jointly evaluated the risk of malnutrition, the olfactory capacity, and cognitive impairment—metrics that require qualified personnel, and are not often measured on a routine basis.

## 4. Materials and Methods

### 4.1. Study Participants

Participants that met the following criteria were included in the study: (1) older adults aged 65 years or older, hospitalized in a tertiary referral hospital in the geriatric clinic, during the period between August 2023 and August 2024; (2) a confirmed diagnosis of long COVID made by the attending physician and registered on medical chart documentation, defined by the presence of a confirmed PCR positive test for SARS-CoV-2 (>3 months previous to the admission) and persistence in symptoms after the acute confirmed infection with SARS-CoV-2 beyond 12 weeks as a continuous, relapsing/remitting, or progressive disease state; and (3) voluntary consent to participate in the study, after explanation and signature of the informed consent. When needed, adaptations to communication were implemented, ensuring hearing aids and glasses were used; making extra efforts to confirm comprehension, simplified language was used, and we allowed ample time for questions. We respected autonomy by assuming capacity unless proven otherwise; when needed, a legal guardian was invited to the discussion, and they provided the signature to participate in the study.

OAs were excluded if they had a previous diagnosis in the electronic medical records related to a psychiatric condition such as bipolar disorder, schizophrenia, a neurological diagnosis of dementia or an important cognitive impairment that impeded participants from answering questionnaires or obtaining study measurements.

The study protocol was designed according to the STROBE [40] and RECORD guidelines for observational studies [41] and submitted for IRB approval before patient recruitment. The study was conducted according to the Declaration of Helsinki [42].

### 4.2. Study Design

This was a cross-sectional study, performed at a geriatric clinic in a tertiary referral hospital. For all participants, data were collected immediately after hospital admission.

### 4.3. Study Measurements

#### 4.3.1. Demographic and Clinical Variables

Demographic and clinical information related to COVID-19 infection and long COVID was obtained by automated retrieval from the institution’s electronic medical records system. Physical examination, assessment of olfactory function, cognitive evaluation, and nutritional evaluation were performed within the first 48 h of patient hospitalization.

#### 4.3.2. Olfactory Functions

To measure olfactory function in our patients, we used the Sniffin’ Stick Test II, which has been previously validated for this purpose [43]. The capacity for odor identification was evaluated using 12 common odors; the identification of <9 odors was defined as functional hyposmia and <6 as anosmia. In each participant, olfactory tests, nutritional status, and cognitive abilities were performed during the same session in a well-ventilated room. First, we evaluated olfactory function, then cognitive abilities, and finally nutritional status.

#### 4.3.3. Cognitive Impairment Evaluation

A geriatric cognitive impairment evaluation of participants was assessed by attending physicians at the geriatric clinic, with validated screening tools routinely used in clinical care, available at the National Institute of Geriatrics website [44]. Cognitive impairment evaluation included the following domains: orientation to time, memory, attention and executive function, assessed with the adapted Spanish version [45] from the previously published Folstein Mini-Mental State Examination (MMSE) [46]. Scores ranged from 0 to 30, with lower scores indicating increasing severity of cognitive impairment. Subjects with a score 0–17 were considered to have CI, scores 18–23 were considered at risk of CI, and individuals with scores ≥24 were considered not to have CI. Functional status was not systematically reassessed by the study team.

#### 4.3.4. Nutritional Assessment

Measurements were obtained by a certified geriatric nutritionist, and included the Mini Nutritional Assessment (MNA^®^), which has been validated for OAs [15]. The MNA includes anthropometric measurements, a global assessment, a dietary questionnaire and a subjective assessment. According to the developers’ instructions, administration of the MNA utilizes a two-step approach: screening and a global assessment. Subsequently, based on the MNA total score, patients are classified as “malnourished” (score < 17), “at risk of malnutrition” (scores 17.5–23.5) or as having a “normal nutritional status” (score > 24). The “global assessment step” of the MNA should only be administered to patients not reaching the screening threshold. Additionally, other measurements were added to the nutritional status including height, weight, mid-arm circumference (MAC) and calf circumference (CC). Weight was measured in kilograms, with participants standing up, using a Tanita BC-558 Ironman scale. Height was measured in centimeters with participants placed under the stadiometer, head facing forward, without shoes, and feet together, using a Seca 213 portable stadiometer. Calf circumference (CC) was measured in centimeters with Lufkin anthropometric tape, at the point where the calf acquires greater volume between the ankle and knee. The mid-arm circumference (MAC) was measured in centimeters with Lufkin’s anthropometric tape, performed on the arm bent in a 90° position and with a tape measure at the midpoint between the bony points of the acromion and olecranon.

#### 4.3.5. Statistical Analysis

Categorical variables were presented as frequencies and percentages; continuous variables were expressed as mean ± standard deviation (SD). Inferences for categorical and continuous variables were established with the chi squared and Student’s *T*-test respectively. The exact Fisher’s test was used for small cell sizes (<5) in tables. For analysis of continuous dependent variables, we used single and multiple linear regression models to establish the association of risk factors for cognitive impairment in long COVID and for the decline in olfactory function. Spearman correlation analysis was conducted to assess the strength and direction of associations between changes in olfactory function (sniffing test) and related continuous variables. A *p* value of <0.05 was considered significant; we used the software Stata (version 19, StataCorp LLC, College Station, TX) for calculations.

## 5. Conclusions

In conclusion, our study presents suggestive risk factors for olfactory decline and CI for OAs in a sex-specific manner. The univariate analysis showed self-reported memory problems to be associated with CI for both sexes, while palpitations and dyspnea where only associated with CI in men. While nutritional status measurements seem to impact the development of CI majorly in women, this association was not conserved after an adjusted regression analysis. The only variable that remained significantly associated with CI in women was the sniffing test, suggesting that the routine evaluation of olfaction should be included as part of the cognition status in female OAs. For men, during the single variable association analysis, renal affection and the number of days with long COVID were associated with CI; however, after adjusting, none remained significant. For an altered sense of smell, the only variable that remained with significance was fewer days with long COVID but only for women. These findings should be confirmed in larger, long-term, multicenter studies with the inclusion of inflammatory biomarkers, which will allow us to define better treatment strategies to prevent CI and its associated deleterious outcomes in OAs.

## Figures and Tables

**Table 1:** Sociodemographic and clinical characteristics of older adults with long COVID divided by sex.

Variable	Women (n = 23)	Men (n = 22)	*p* Value
Nutritional Parameters, mean ± SD, or median (ICR)			
Age, y	74.2 ± 5.6	77.6 ± 6.8	0.079
Weight, kg	65.9 ± 17.1	64.7 ± 14.1	0.794
Height, mt	1.5 ± 0.1	1.7 ± 0.1	<0.001 *
BMI	27.7 (21.8–32.2)	21.9 (18.9–26.1)	0.017 *
CC	32.0 (28.5–35.0)	28.9 (26.3–36.6)	0.063
MAC	28.0 (23.5–32.0)	25.4 (23.4–30.3)	0.294
Obesity, No. (%)	10 (44)	4 (18)	0.067
Comorbidities, No. (%)			
Basic education	20 (87)	19 (86)	0.820
Diabetes	12 (53)	6 (27)	0.088
Hypertension	14 (61)	11 (50)	0.463
Kidney disease	5 (22)	5 (23)	0.936
COPD	8 (34)	8 (36)	0.912
>3 comorbidities	17 (74)	9 (41)	0.025 *
Smoker	2 (9)	4 (18)	0.349
Cardiovascular	16 (70)	19 (86)	0.175
Autoimmunity	4 (17)	3 (14)	0.728
Long COVID Symptoms, No. (%)			
Anosmia	19 (83)	20 (91)	0.413
Ageusia	13 (57)	12 (55)	0.894
Fatigue	19 (83)	22 (100)	0.040 *
Memory problems	17 (74)	16 (73)	0.928
Insomnia	16 (70)	19 (86)	0.175
Cough	11 (48)	15 (68)	0.167
Dyspnea	9 (39)	15 (68)	0.051
Palpitations	0 (0)	6 (27)	0.007 *
Brainfog	5 (22)	5 (23)	0.936
Dizziness	3 (13)	3 (14)	0.953
Depression	10 (44)	6 (27)	0.256
Myalgias	8 (35)	5 (23)	0.372
COVID Prevention and Severity, No. (%) or median (ICR)			
Vaccination	15 (65)	14 (64)	0.912
Hospitalization	12 (52)	12 (55)	0.873
Supplementary O_2_	14 (60)	18 (82)	0.325
Intubation	2 (9)	0 (0)	0.325
In-hospital, days	5.0 (0–11)	9.5 (0–22)	0.477
COVID reinfection	3 (13)	0 (0)	0.080
Long COVID, days	88 (75–212)	120 (88–221)	0.768
Tests, median (ICR)			
MNA	17.0 (15.5–23)	18.5 (15.5–22.5)	0.865
MMSE	20.0 (16.0–25.0)	23.5 (20.0–25.0)	0.291
Sniffing test	9.0 (6.0–10.0)	7.5 (5.0–10.0)	0.353
Older adult symptoms, No. (%)			
Frailty	13 (57)	16 (72)	0.256
Sarcopenia	4 (17)	8 (36)	0.150
Malnourishment	18 (50)	18 (50)	0.766

BMI: Body mass index; CC: calf circumference; MAC: mean arm circumference; COPD: Chronic Obstructive Pulmonary Disease; MNA: Mini-Nutritional Assessment; MMSE: Mini-Mental State Examination; ICR: interquartile range. * Statistical significance (*p* < 0.05).

**Table 2:** Gender-specific clinical parameters associated with cognitive impairment (CI) assessed with the Mini-Mental State Examination (MMSE).

Variable	Women (n = 23)		*p* Value	Men (n = 22)		*p* Value
	Without CI (n = 6)	Risk of CI or CI (n = 17)		Without CI (n = 9)	Risk of CI or CI (n = 13)	
Nutritional parameters, mean ± SD, or median (ICR)						
Age, y	73.1 ± 3.7	74.7 ± 6.2	0.574	77.3 ± 6.7	77.8 ± 7.1	0.889
Weight, kg	55.8 ± 22.2	69.5 ± 14.0	0.091	64.1 ± 16.0	65.1 ± 13.3	0.879
Height, mt	1.5 ± 0.1	1.5 ± 0.1	0.776	1.7 ± 0.1	1.7 ± 0.0	0.801
BMI	23.3 ± 9.3	29.5 ± 6.3	0.080	23.2 ± 5.7	23.3 ± 4.5	0.959
CC	28.8 ± 4.4	33.0 ± 3.9	0.039 *	29.3 ± 5.6	29.4 ± 4.1	0.961
MAC	24.3 ± 4.8	30.2 ± 5.5	0.031 *	28.4 ± 8.0	25.7 ± 3.8	0.304
Obesity, No. (%)	1 (10)	9 (90)	0.022 *	2 (22)	2 (22)	0.683
Comorbidities, No. (%)						
Basic education	6 (30)	14 (70)	0.270	7 (39)	11 (61)	0.683
Diabetes	2 (17)	10 (83)	0.275	3 (50)	3 (50)	0.477
Hypertension	3 (50)	11 (65)	0.643	5 (56)	6 (46)	0.665
Kidney disease	3 (50)	2 (12)	0.089	0 (0)	5 (38)	0.049 *
COPD	3 (50)	5 (29)	0.334	2 (22)	6 (46)	0.246
Smoker	3 (50)	13 (76)	0.420	1 (11)	3 (23)	0.474
Ex-smoker	1 (17)	2 (12)	0.616	3 (33)	5 (38)	0.584
Cardiovascular	3 (50)	13 (76)	0.226	7 (78)	12 (92)	0.329
Autoimmunity	1 (17)	3 (18)	0.957	0 (0)	3 (23)	0.121
Long COVID Symptoms, No. (%)						
Anosmia	6 (32)	13 (68)	0.191	7 (35)	13 (65)	0.075
Ageusia	4 (30)	9 (70)	0.560	5 (42)	7 (58)	0.937
Fatigue	6 (32)	13 (68)	0.191	9 (41)	13 (59)	0.138
Memory problems	2 (12)	15 (88)	0.008 *	4 (25)	12 (75)	0.013 *
Insomnia	4 (25)	12 (75)	0.858	8 (42)	11 (58)	0.774
Cough	1 (17)	10 (59)	0.076	7 (78)	8 (62)	0.421
Dyspnea	4 (67)	5 (29)	0.108	8 (89)	7 (54)	0.083
Palpitations	0 (0)	0 (0)	-	5 (56)	1 (8)	0.003 *
Brainfog	2 (33)	3 (18)	0.423	2 (22)	3 (23)	0.013 *
Dizziness	0 (0)	3 (18)	0.270	1 (11)	2 (15)	0.774
Depression	4 (67)	6 (35)	0.183	2 (22)	4 (31)	0.658
Myalgias	3 (50)	5 (29)	0.363	3 (33)	2 (15)	0.323
COVID Prevention and Severity, No. (%) or mean ± SD						
Vaccination	4 (67)	11 (65)	0.931	4 (44)	10 (77)	0.119
Hospitalization	5 (42)	7 (58)	0.076	4 (33)	8 (66)	0.429
Supplementary O_2_	4 (33)	10 (67)	0.278	8 (42)	10 (58)	0.774
Intubation	1 (50)	1 (50)	0.420	0	0	-
In-hospital, days	34.3 ± 48.6	5.5 ± 6.3	0.021 *	27 ± 43.8	13.9 ± 18.5	0.345
COVID reinfection	1 (33)	2 (66)	0.759	0	0	-
Long COVID, days	182.8 ± 113.5	230.4 ± 215.0	0.614	137.8 ± 40.6	301.8 ± 219.3	0.039 *
Tests, mean ± SD						
MNA	14.4 ± 2.0	20.2 ± 4.5	<0.007 *	16.6 ± 5.0	19.6 ± 4.2	0.134
MMSE	27.1 ± 1.9	18.1 ± 3.9	<0.001 *	26.3 ± 2.1	19.3 ± 4.4	0.003 *
Sniffing test	8.0 ± 1.8	8.0 ± 3.1	1.0	8.1 ± 3.2	6.6 ± 2.2	0.216
Older adults syndromes, No. (%)						
Frailty	2 (33)	11 (65)	0.523	5 (56)	11 (85)	0.416
Sarcopenia	1 (17)	3 (18)	0.309	2 (22)	6 (46)	0.264
Biochemical parameters, mean ± SD						
Hb	12.0 ± 4.3	12.1 ± 2.8	0.961	12.2 ± 2.2	11.1 ± 4.0	0.453
Leukocytes	12.5 ± 9.0	9.4 ± 5.9	0.350	9.4 ± 4.1	9.8 ± 4.7	0.842
Lymphocytes	1.7 ± 0.9	1.5 ± 0.9	0.616	1.8 ± 0.8	1.2 ± 0.5	0.065
Neutrophiles	10.8 ± 9.6	7.2 ± 6.2	0.335	6.5 ± 4.3	7.1 ± 4.0	0.750
Platelets	365.4 ± 132.5	207.3 ± 61	0.001 *	226.4 ± 91.8	274.6 ± 135.4	0.365
Creatinine	0.7 ± 0.2	1.1 ± 0.9	0.329	1.5 ± 1.6	1.0 ± 0.6	0.334
Urea	42.8 ± 14.7	60.5 ± 56.2	0.461	63.4 ± 64.9	56.8 ± 36.8	0.764
LDH	250 ± 73.5	206 ± 110.4	0.613	181.8 ± 77.7	220.0 ± 138.0	0.597
AST	30.8 ± 8.7	31.3 ± 26.3	0.466	21 ± 9.8	17.4 ± 11.1	0.514
ALT	24.8 ± 8.6	19.4 ± 8.9	0.268	32.8 ± 40.7	12.5 ± 7.2	0.104

CI: cognitive impairment; BMI: body mass index; CC: calf circumference; MAC: mean arm circumference; COPD: Chronic Obstructive Pulmonary Disease; MNA: Mini-Nutritional Assessment; MMSE: Mini-Mental State Examination; ICR: interquartile range. * Statistical significance (*p* < 0.05).

**Table 3:** Coefficients of variation in a multivariate analysis for CI, assessed with the Folstein Mini-Mental State Examination (MMSE), by sex.

**Women**				
Variable	Coefficient	*R* ^2^	*p*	(95% CI)
Age, yr	−0.488	0.566	0.075	−1.036–0.588
MNA	−0.317		0.370	−1.057–0.424
BMI (kg/m^2^)	0.073		0.663	−0.284–0.430
CC	−0.3167		0.482	−1.267–0.633
MAC	−0.428		0.228	−1.163–0.307
Long COVID, days	0.003		0.398	−0.024–0.010
Sniffing test	1.664		0.009 *	0.501–2.828
Frailty	−6.435		0.065	−13.330–0.461
Sarcopenia	−2.630		0.397	−9.145–3.887
**Men**				
Age, yr	−0.164	0.518	0.423	−0.593–0.266
MNA	−0.315		0.338	−1.001–0.372
BMI (kg/m^2^)	−0.868		0.113	−1.973–0.041
CC	0.067		0.899	−0.794–0.927
MAC	0.740		0.111	−0.060–1.420
Long COVID, days	−0.010		0.093	−0.022–0.002
Sniffing test	0.654		0.225	−0.460–1.768
Frailty	−2.781		0.237	−7.650–2.087
Sarcopenia	0.398		0.914	−7.442–8.239

CI: cognitive impairment; MNA: Mini Nutritional Assessment; BMI: body mass index; CC: calf circumference; MAC: mid-arm circumference. * Statistical significance (*p* < 0.05).

**Table 4:** Coefficients of variation in univariate analysis for altered sense of smell.

Variable	Coefficient	*R* ^2^	*p*	(95% CI)
Age, yr	−0.178	0.168	0.005 *	−0.299–0.056
Male	−0.773	0.020	0.353	−2.433–0.888
MNA	0.133	0.051	0.135	−0.043–0.309
BMI	0.142	0.120	0.020 *	0.024–0.261
Obesity	1.895	0.121	0.019 *	0.322–3.468
CC	0.272	0.212	0.001 *	0.111–0.435
MAC	0.229	0.238	0.001 *	0.103–0.356
Long COVID, days	−0.005	0.123	0.018 *	−0.009–−0.001
COVID reinfection	−1.381	0.016	0.408	−4.716–1.954

MNA: Mini Nutritional Assessment, BMI: body mass index, CC: calf circumference, MAC: mid-arm circumference. * Statistical significance (*p* < 0.05).

**Table 5:** Coefficients of variation in a multivariate analysis for altered sense of smell, assessed with the sniffing test, by sex.

**Women**				
Variable	Coefficient	*R* ^2^	*p*	(95% CI)
Age, yr	−0.129	0.472	0.227	−0.347–0.089
MNA	0.065		0.693	−0.276–0.406
BMI (Kg/m^2^)	0.318		0.714	−0.149–0.213
CC	0.240		0.561	−0.400–0.225
MAC	−0.088		0.223	−0.163–0.641
Long COVID, days	−0.008		0.014 *	−0.014–−0.001
**Men**				
Age, yr	−0.101	0.453	0.348	−0.324–0.121
MNA	−0.111		0.467	−0.429–0.206
BMI (Kg/m^2^)	0.026		0.916	−0.488–0.534
CC	0.129		0.374	−0.341–0.599
MAC	0.157		0.569	−0.209–0.523
Long COVID, days	−0.003		0.391	−0.009–0.004

CI: cognitive impairment, MNA: Mini Nutritional Assessment, BMI: body mass index, CC: calf circumference, MAC: mid-arm circumference. * Statistical significance (*p* < 0.05).

## Data Availability

The datasets used and/or analyzed during the current study are available from the corresponding author upon reasonable request.

## Abbreviations

The following abbreviations are used in this manuscript:

BMI	Body mass index
CC	Calf circumference
MAC	Mid-arm circumference
MMSE	Mini-Mental State Examination
OA	Older adults
